# Characterization of microchannel plate detector response for the detection of native multiply charged high mass single ions in orthogonal‐time‐of‐flight mass spectrometry using a Timepix detector

**DOI:** 10.1002/jms.4820

**Published:** 2022-03-28

**Authors:** Anjusha Mathew, Gert B. Eijkel, Ian G. M. Anthony, Shane R. Ellis, Ron M. A. Heeren

**Affiliations:** ^1^ Maastricht MultiModal Molecular Imaging (M4i) Institute, Division of Imaging Mass Spectrometry (IMS) Maastricht University Maastricht The Netherlands; ^2^ Molecular Horizons and School of Chemistry and Molecular Bioscience University of Wollongong Wollongong NSW Australia

**Keywords:** microchannel plate detector, native mass spectrometry, single ion imaging, time‐of‐flight mass spectrometry, Timepix detector

## Abstract

Time‐of‐flight (TOF) systems are one of the most widely used mass analyzers in native mass spectrometry (nMS) for the analysis of non‐covalent multiply charged bio‐macromolecular assemblies (MMAs). Typically, microchannel plates (MCPs) are employed for high mass native ion detection in TOF MS. MCPs are well known for their reduced detection efficiency when impinged by large slow moving ions. Here, a position‐ and time‐sensitive Timepix (TPX) detector has been added to the back of a dual MCP stack to study the key factors that affect MCP performance for MMA ions generated by nMS. The footprint size of the secondary electron cloud generated by the MCP on the TPX for each individual ion event is analyzed as a measure of MCP performance at each mass‐to‐charge (*m/z*) value and resulted in a Poisson distribution. This allowed us to investigate the dependency of ion mass, ion charge, ion velocity, acceleration voltage, and MCP bias voltage on MCP response in the high mass low velocity regime. The study of measurement ranges; ion mass = 195 to 802,000 Da, ion velocity = 8.4 to 67.4 km/s, and ion charge = 1+ to 72+, extended the previously examined mass range and characterized MCP performance for multiply charged species. We derived a MCP performance equation based on two independent ion properties, ion mass and charge, from these results, which enables rapid MCP tuning for single MMA ion detection.

## 1 INTRODUCTION

The introduction of soft ionization techniques, such as electrospray ionization (ESI)^1^ and matrix‐assisted laser desorption/ionization (MALDI)^2^ in late 1980s, allowed the mass analysis of intact biomolecules ranging from several daltons to few megadaltons.^3^, ^4^, ^5^ The utilization of nanoESI source^6^, ^7^ and volatile high ionic strength solvents,^8^, ^9^ and advancements in mass spectrometry (MS) instrumentation^10^, ^11^, ^12^ further extended the mass range to several megadaltons, by enabling the ionization and analysis of non‐covalent bio‐macromolecular assemblies (MMAs) in their pseudo‐native state,^13^, ^14^, ^15^ where quaternary structure is retained, a method referred to as native MS (nMS).^16^

Time‐of‐flight (TOF) MS is one of the most commonly used mass analyzers for high mass detection due to its unlimited theoretical mass range, high sensitivity, and speed of analysis.^17^, ^18^ Ion detection in TOF MS is traditionally accomplished using microchannel plates (MCPs) because of their high gain, fast response, and large active area.^19^, ^20^, ^21^ However, MCP detectors suffer from a reduced detection efficiency when impinged by large slowly moving ions.^22^, ^23^, ^24^, ^25^, ^26^ Hence, the key parameters affecting the detection of high mass non‐covalent ions generated by nMS must be better understood. Here, we conducted a detailed study to gain insight into the influence of critical ion and ion optical parameters on MCP detector performance for high mass multiply charged slow moving protein/protein complex ions, using the spatially and temporally resolved detection of individual ion events.

In previous studies, the performance of the MCP has been examined for the detection of singly charged biomolecules of mass up to 290 kDa, by measuring the secondary electron yield (*γ*, average number of electrons produced within the MCP per initial ion impact event) and/or detection efficiency (*ε*, probability of generation of one or more secondary electrons during the initial ion impact event), by comparing the ion counts at different acceleration voltages,^22^ using a superconducting tunnel junction (STJ) detector^24^ or an inductive charge detector (ICD)^25^, ^26^ in parallel with MCP detector. Secondary electron emission from the impacts of low velocity macro‐ions of *m/z* < 100,000 Da from various conducting surfaces has also been studied.^27^, ^28^, ^29^, ^30^, ^31^ In this work, we have added an active pixelated detector (Timepix [TPX]) to the back of a dual MCP stack on a modified orthogonal reflectron TOF (O/R‐TOF) MS (LCT) equipped with a nanoESI source.^32^ With this unique setup, we analyzed the individual footprints of secondary electron clouds generated by the MCP on the TPX detector. As the size of each electron cloud depends on the number of electrons produced from an individual ion impact due to its space‐charge‐driven expansion, this allowed us the study of MCP amplification as a function various parameters for multiply charged MMA ions of molecular weights up to 802 kDa.

The TPX is a position‐ and time‐sensitive charge detector consisting of a 512 × 512 pixel array with each pixel capable of recording both the arrival time and impact coordinates of impinging particles.^33^ Despite the fact that TPX technology has its origin in high energy physics,^34^, ^35^, ^36^, ^37^ the integration of TPX with MCP amplifier allowed the detection of low energy particles,^38^, ^39^, ^40^, ^41^ which extended its scope to MS applications.^21^ MCP–TPX assembles have been used in MS with the goals (i) to improve the spatial resolution and throughput of MS imaging^42^, ^43^, ^44^, ^45^; (ii) to investigate the ion transport properties through different ion optical elements of MS^32^, ^46^, ^47^; and (iii) for the enhanced detection of high mass ions.^32^, ^48^, ^49^ A previous study from our group conducted on the same TPX equipped LCT system has demonstrated the capability of TPX to detect non‐covalent protein complexes and to image single ion events.^32^

We present a detailed characterization of MCP response for high mass multiple charged non‐covalent species using the single ion imaging capability of TPX detector. We analyzed the electron cloud footprints on the TPX correspond to each mass‐to‐charge (*m/z*) value from a set of 16 samples that encompasses the following measurement ranges: ion mass = 195 to 802,000 Da, TOF = 16 to 155 μs, ion velocity = 8.4 to 67.4 km/s, ion charge = 1+ to 72+, *m/z* = 195 to 12,500 Da, and ion energy = 4.6 to 331.2 keV. The main objective of this study is to develop a better understanding of the dependency of ion mass, ion velocity, ion charge, acceleration voltage, and MCP bias voltage on MCP performance in high mass low velocity regime.

## 2 MATERIALS AND METHODS

### 2.1 Materials

Ubiquitin (8.6 kDa) from bovine erythrocytes, cytochrome C (12.4 kDa) from equine heart, myoglobin (17.6 kDa) from equine heart, carbonic anhydrase (29 kDa) from bovine erythrocytes, bovine serum albumin (BSA; 66.4 kDa), conalbumin (77 kDa) from chicken egg white, concanavalin A (102 kDa) from *Canavalia ensiformis*, alcohol dehydrogenase from *Saccharomyces cerevisiae* (147.5 kDa), trastuzumab monoclonal antibody (148 kDa), beta amylase (β amylase; 223.8 kDa) from sweet potato, pyruvate kinase (232 kDa) from rabbit muscle, apoferritin (480 kDa) from equine spleen, chaperonin 60 (GroEL; ~802 kDa) from *Escherichia coli*, ammonium acetate, tris acetate, potassium chloride, ethylenediaminetetraacetic acid (EDTA), adenosine‐5′‐triphosphate (ATP), magnesium chloride, ammonium hydroxide, and acetic acid were all purchased from Sigma‐Aldrich (Zwijndrecht, The Netherlands). Pierce™ LTQ ESI positive ion calibration solution (195 to 1522 Da) and cesium iodide (CsI; 392.7 to 11,304 Da) were purchased from Thermo Fisher Scientific, The Netherlands. Glu‐fibropeptide B (1.6 kDa) was obtained from Waters, The Netherlands. Methanol, acetone, isopropanol, and LC–MS grade water were purchased from Biosolve (Valkenswaard, The Netherlands).

### 2.2 Sample preparation

Ubiquitin, cytochrome C, myoglobin, carbonic anhydrase, BSA, conalbumin, concanavalin A, alcohol dehydrogenase, trastuzumab monoclonal antibody, and β amylase were dissolved to a stock concentration of 100 μM in LC–MS grade water. These samples and aqueous solutions of pyruvate kinase and apoferritin were buffer exchanged with 200 mM ammonium acetate at pH 6.8 using 3/10/30 kDa molecular weight cutoff (MWCO) Amicon Ultra centrifugal filter (Millipore, Merck KGaA, Germany) to a final monomer concentration of 5–30 μM. The preparation of GroEL was performed as described previously.^32^ Glu‐fibropeptide B was dissolved in 200 mM ammonium acetate (pH 6.8) to a concentration of 5 μM. CsI was prepared as 2 mg/ml solution in 1:1 isopropanol:water (v:v).

### 2.3 Mass spectrometer and detection system

All experiments were performed on a modified LCT nESI‐O‐TOF mass spectrometer (Micromass, Manchester, UK) equipped with a TPX detector that has recently been described in detail in reference.^32^ Samples were introduced into the mass spectrometer using homemade gold‐coated needles via an in‐house built static nanoESI source. Ions are transferred into an orthogonal acceleration TOF mass analyzer for TOF separation via two differentially pumped hexapole RF lenses. The instrument has been modified for improved transmission of high *m/z* ions as described previously.^32^

The detector assembly consist of an MCP–TPX system. The dual chevron MCP stack (Type No. F4294‐07, Hamamatsu Photonics, Japan) employed in this study has the following properties: 27 mm effective diameter, 0.4 mm plate thickness, 12° bias angle, 12.5 μm channel center‐to‐center spacing, 10 μm channel diameter, and 4–20 μA strip current. A bare TPX quad application‐specific integrated circuit (ASIC) is positioned 2 mm behind the dual MCP stack of the LCT to detect the secondary electron clouds emitted from the MCP during an ion event. The TPX is a position‐ and time‐sensitive charge detector that consists of 512 × 512 pixel array with a pixel pitch of 55 μm, in which each pixel is a single stop time‐to‐digital converter (TDC) that registers an event once the input charge of a given pixel exceeds a certain threshold (equivalent to ~600 electrons).^33^ In this study, TPX has been operated in time‐of‐arrival (TOA) mode, in which the time of activation of each pixel is measured along with pixel coordinates with respect to an external trigger.^43^ The TPX was triggered at a rate of 30–40 Hz using a down‐sampled version of the main trigger pulse that starts the orthogonal acceleration in the pusher, via a digital pulse and delay generator (DG535, Stanford Research Systems). All experiments were performed using a 20 ns TPX clock width, corresponding to a maximum measurement window of 236.2 μs for each TOF cycle. All data reported in this paper were recorded using the following voltage settings: DC offset 1: 10 V, DC offset 2: 6 V, ion energy: 10 V, aperture: 20 V, TOF tube: 4600 V, reflectron: 1000 V, MCP gain: 1600 V, and TPX: −2200 V unless stated otherwise. This resulted in typical flight times between 16 and 155 μs. Five microliters of each sample was loaded into the nano‐ESI needle and a spray voltage of 0.9–1.8 kV was applied. All ion optics parameters except the TPX and spray voltages were defined via MassLynx V4.1 software (Waters, Wilmslow, UK). External power supplies (FuG Elektronik GmbH, Schechen, Germany) were used to provide a voltage offset to the TPX relative to the back MCP and spray voltage.

All samples were characterized using a Q Exactive UHMR Hybrid Quadrupole‐Orbitrap mass spectrometer (Thermo Fisher Scientific, Bremen, Germany) prior to the measurements on the LCT‐TPX system for the TOF to *m/z* conversion.

### 2.4 Data acquisition and analysis

The SoPhy (Software for Physics) software package Version 1.5.2 was used for TPX chip control and data acquisition (Amsterdam Scientific Instruments, Amsterdam, The Netherlands). A total of 5000–10,000 TOF cycles (frames) were collected and summed for each dataset. SoPhy generates a binary 512 × 512 frame for each TOF cycle. Every *x*–*y* position (pixel) in the frame contains information on the number of clock counts passed since the start of the TOF cycle and the arrival of sufficient charge to trigger that pixel. One hundred frames are bundled in a zipped output file. An in‐house developed algorithm written in MATLAB (R2014a, MathWorks Inc., Natick, MA, USA) was used to convert the data to NetCDF format and to select sub‐frames from frames and TOF ranges. The MATLAB functions “bwlabel” and “regionprops” were used to detect and measure the properties of connected areas (pixel clusters) in 2D binary sub‐frames.

## 3 RESULTS AND DISCUSSION

### 3.1 Single ion imaging using MCP–TPX assembly

MCP assemblies are particle amplifiers that intensify low energy particles by the multiplication of electrons via secondary emission and amplification. The amount of secondary electrons generated within the dual MCP stack is related to the impinging particle properties and bias voltage across the MCP plates (MCP bias voltage). Zero to *n* number of electrons may eject from the front MCP plate upon the impact of a single ion on one of the microchannels, and these electrons generate more electrons and are accelerated to the back MCP plate based on the MCP bias voltage. Under typical MS conditions, the amplification factor is often nominally in the order of 10^5^–10^6^. The secondary electron multiplicity is described by a Poisson distribution given by

(1) $$P_{n}=\frac{\gamma^{n}e^{-\gamma }}{n!}$$

where *P*_*n*_ is the probability of emitting *n* electrons from the MCP and *γ* is the average number of electrons emitted per initial ion impact, known as secondary electron yield.^25^ The detection efficiency, *ε*, is defined as the probability of emitting at least one electron from a single ion impact and given by

(2) $$\varepsilon =1-P_{0}=1-e^{-\gamma }$$

where *P*_0_ is the probability of not emitting any electrons and calculated as *e* ^−*γ*^ from Equation (1).

In TOF MS, all ions are accelerated with the same kinetic energy per charge because they are all subjected to the same acceleration voltage, $\mathrm{zeV}=1/2mv^{2}$, where *ze*, *V*, *m*, and *v* are ion charge (*e* is the charge of an electron and *z* is the number of charges on the ion), acceleration voltage, ion mass, and ion velocity, respectively. High *m/z* ions impinge the MCP detector with a lower velocity, resulting in reduced *γ*, making it harder to detect large slow moving ions.^22^, ^23^, ^24^, ^25^, ^26^

Several groups previously examined the performance of the MCP in high mass low velocity regime by measuring *γ* and/or *ε* with different approaches. Macfarlane's group has calculated *ε* through the following procedure. First, the ion intensity at a high acceleration potential was measured to determine the integrated intensity of particular ions under conditions where *P*(*γ*) = 1 (i.e., *γ* is a large number). Then, the integrated intensity of the same ions was measured at the velocity of interest by reducing the acceleration voltage. Later, the ratio of two intensities was taken for the calculation of *ε*. Note that the ion intensity was derived using the single ion counting technique. The study was conducted for the following measurement range: mass = 86–5734 Da, charge = 1, velocity = 13–32 km/s, and acceleration voltage ≤ 20 kV.^22^ Benner's group measured ion intensity using MCP along with STJ detector, which has 100% detection efficiency, exposing both the detectors simultaneously to nearly identical ion fluxes for the following measurement conditions: mass = 1.3–66 kDa, charge = 1, velocity ≥ 5 km/s, and acceleration voltage = 10–30 kV.^24^ Smith's group calculated *γ* by detecting ions in parallel using both an in‐line non‐destructive ICD that provides an absolute measure of the number of ions and an MCP detector, for the following conditions: mass = 1–290 kDa, charge = ±1, velocity = 3.5–68 km/s, and acceleration voltage = 10–25 kV.^25^, ^26^ In this work, a time‐ and position‐sensitive TPX detector has been combined with a dual MCP stack for the MCP characterization and thus measures the arrival time and size of the emitted electron pulses that span multiple pixels (Figure S1) but correspond to individual ion events.

**FIGURE 1:**
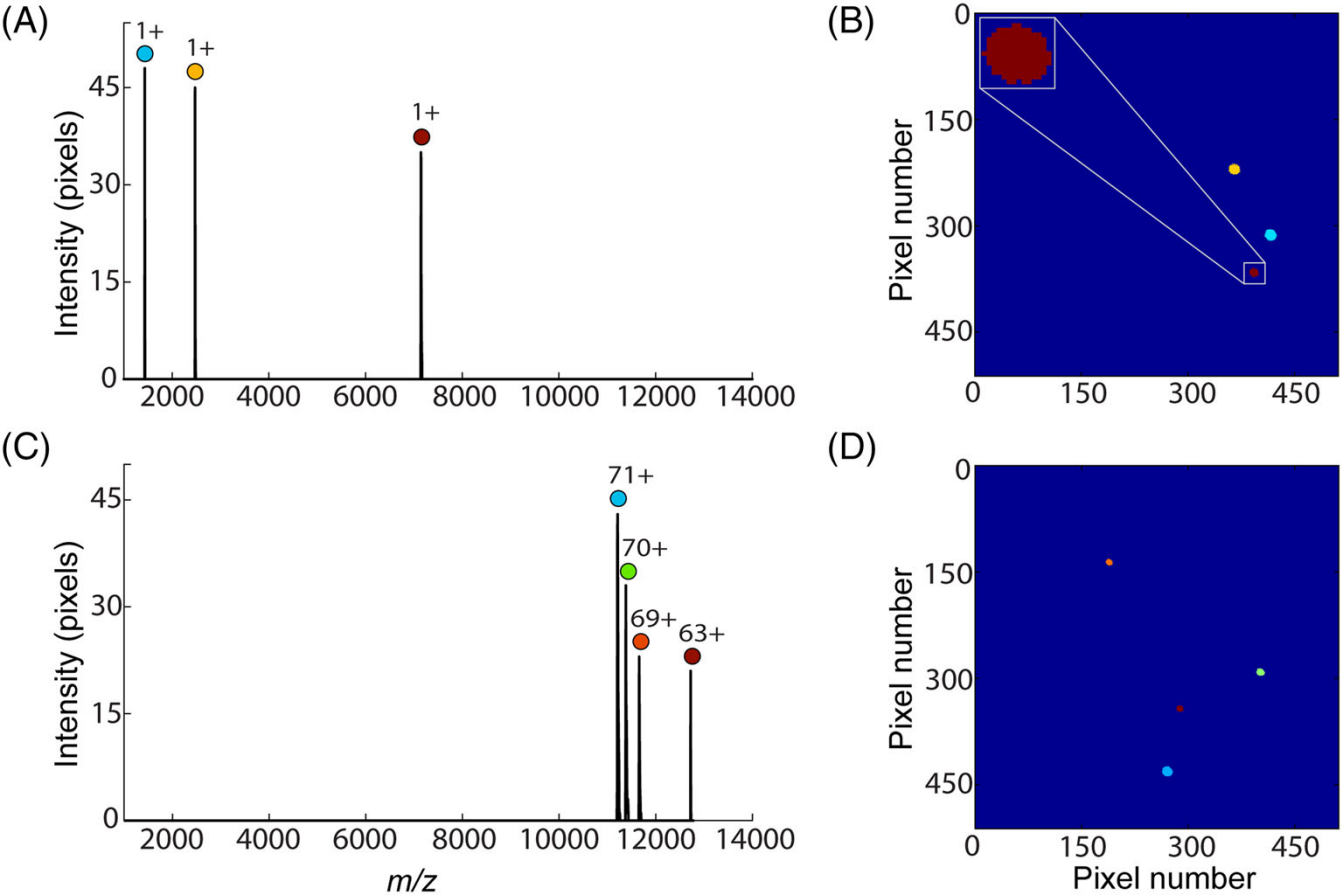
(A) Mass spectrum and (B) TPX image acquired from a single TOF cycle using cesium iodide mix (mass range: 392 to 11,304 Da, charge = 1). (C) Mass spectrum and (D) TPX image acquired from a second single TOF cycle by spraying GroEL (mass: 802 kDa, charge range: 63+ to 72+) under native conditions. The *y*‐axis in (A) and (C) represents the number of TPX pixels activated for each ion event. Each color in the single frame TPX image corresponds to a different *m/z* or time‐of‐arrival

**FIGURE 2:**
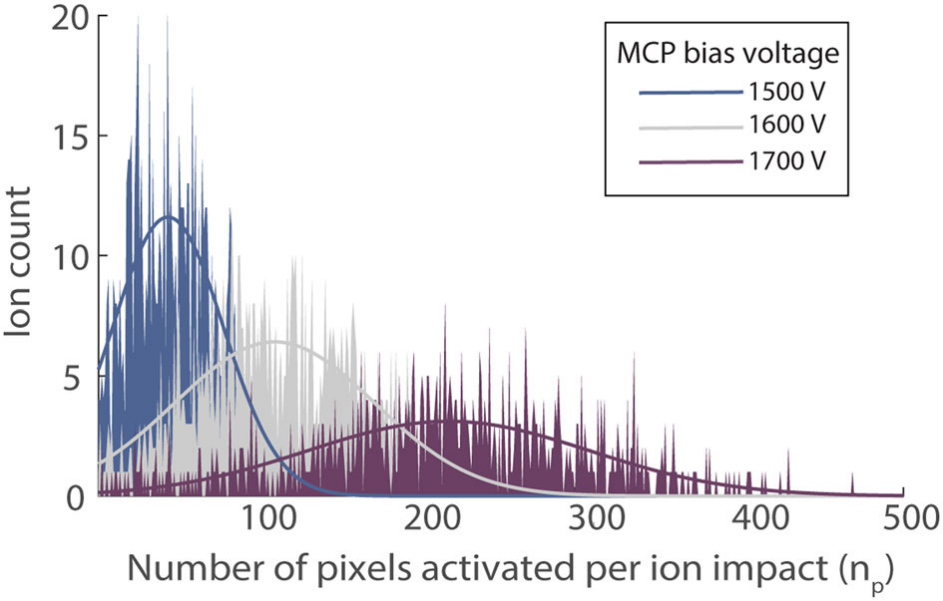
Distribution of pixel cluster area (in pixels, *n*_p_) of 930 cytochrome C [M + 7H]^7+^ ions from 5000 TOF cycles acquired for different MCP bias voltages and corresponding Gaussian fits

The detection in TPX is frame based, and each frame corresponds to a single TOF cycle. Figure 1A,B shows the single frame spectrum and image generated by spraying CsI mix (mass range: 392 to 11,304 Da, charge = 1), and Figure 1C,D corresponds to the single frame spectrum and image of GroEL (mass: 802 kDa, charge range: 63+ to 72+). Each impact event in the single frame image corresponds to the electron footprint of single ion event at the MCP and is associated with a single peak in the mass spectrum. The number of pixels activated by each ion event (*n*_p_) is related to the number of electrons emitted from the MCP, which in turn is related to the efficiency of the initial ion‐to‐electron conversion, amplification steps through the channels of MCP, and the space‐charge‐driven expansion of the electron pulse between the MCP and TPX. In this work, we have measured *n*_p_ instead of *γ*/*ε* to investigate the dependency of ion and voltage parameters on the MCP detector response. Figure 1 suggests that *n*_p_ increases or MCP response improves with an increase in ion charge and decrease in ion mass. However, it is hard to draw a conclusion only by analyzing a single frame. Hence, we have analyzed *n*_p_ corresponds to a single *m/z* or TOA from all the frames. This produces a Poisson statistics (Equation 1) as expected. Figure 2 shows the distribution of *n*_p_ of ∼930 cytochrome C [M + 7H]^7+^ (*m/z =* 1766.7) ions from 5000 TOF cycles acquired for different MCP bias voltages and corresponding Gaussian fits. A higher MCP bias voltage produces more secondary electrons on average for a single ion event. This in turn leads to higher columbic repulsion of the electron pulse as it travels towards the TPX and thus results in a greater number of active pixels. The effect of MCP bias voltage on the detection efficiency will be discussed in more detail later in this article.

### 3.2 Influence of ion properties on MCP performance

In this section, single ion imaging capability of TPX has been utilized to study the influence of ion properties on ion to electron conversion factor and thus the MCP response. *γ* is generally expressed as a function of two dependent parameters, ion mass and velocity,^24^, ^25^, ^26^ given by

(3) $$\gamma =k_{0}m^{a}v^{b}$$

where the *k*_0_ is constant of proportionality, and *a* and *b* are fit parameters. Combining Equation (3) with basic TOF MS equation

(4) $$v=\sqrt{\frac{2\mathrm{zeV}}{m}}\;$$

yields the relation

(5) $$\gamma =k_{0}m^{\frac{2a-b}{2}}{\left(2\mathrm{zeV}\right)}^{\frac{b}{2}}$$

Equation (5) can be rewritten as a function of two independent parameters, ion mass and charge, at a given acceleration voltage as

(6) $$\gamma =k_{1}m^{c}z^{d}$$

where *k*_1_ = *k*_0_(2*eV*)^*b*/2^, *c* = (2*a* − *b*)/2, and *d* = *b*/2.

In this study, instead of calculating *γ*, we have measured the mean *n*_p_ (*μ*) that corresponds to each *m/z* or TOA, which is proportional to *γ*. This is done by spraying 16 samples that encompasses the following measurement range: *m* = 195 to 802,000 Da, TOF = 16 to 155 μs, *v* = 8.4 to 67.4 km/s, *z* = 1+ to 72+, *m/z* = 195 to 12,500 Da, and ion energy = 4.6 to 331.2 keV under identical ion optical conditions. Figure S2 shows the TOF to *m/z* conversion curve, plotted by comparing LCT measured TOF data with the Orbitrap *m/z* spectrum of each sample. From Equation (4) and calibration curve, *v* can be written as *k*_2_ *TOF* ^−0.99^, where *k*_2_ = (1.142 × 10^6^)*k*_0_. Therefore, Equations (3) and 6 can rewritten to calculate *μ* as

(7) $$\mu =k_{3}m^{c}z^{d}=k_{4}m^{a}{\mathrm{TOF}}^{-0.99b}\;$$

where *k*_3_ = (*μ*/*γ*)*k*_1_ and *k*_4_ = (*μ*/*γ*)*k*_2_.

**FIGURE 3:**
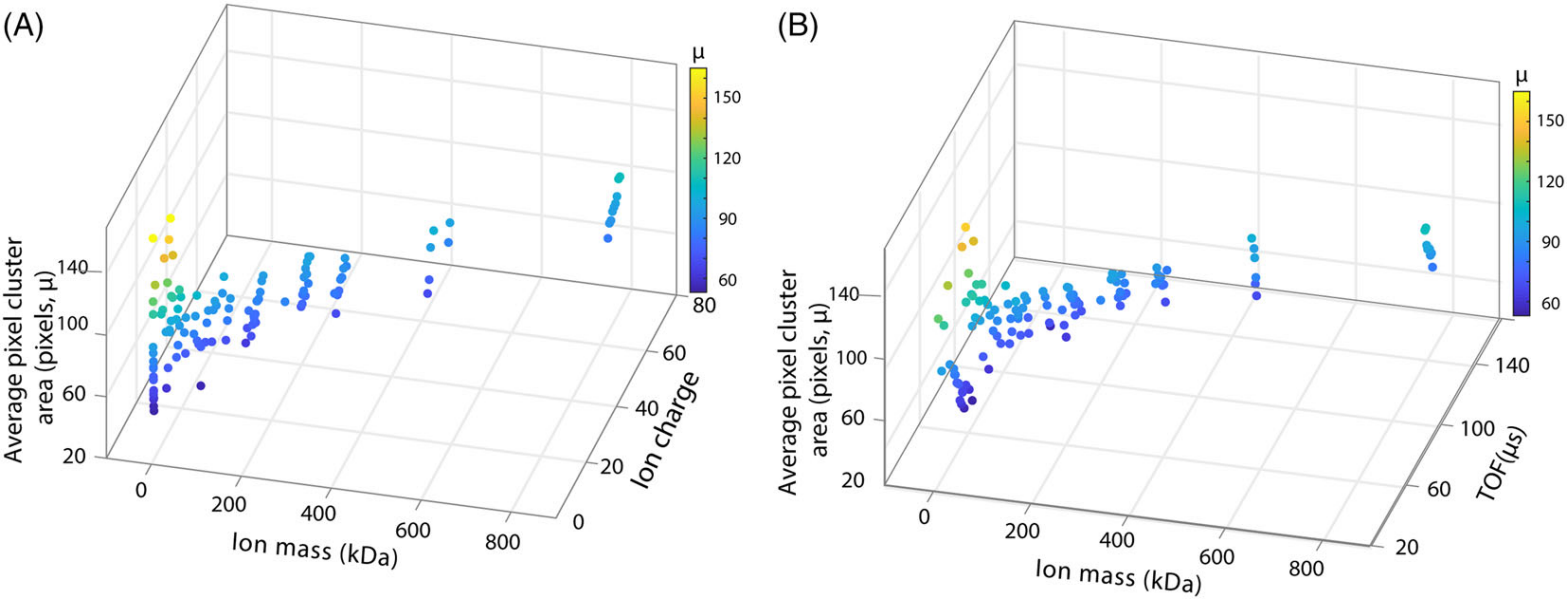
3D scatter plot showing the effect of (A) ion mass and charge and (B) ion mass and TOF on the average pixel cluster area (in pixels, *μ*). All the data were acquired using the following conditions: sample cone: 20–190 V, extraction cone: 10–80 V, RF lens: 200–2000 V, DC offset 1: 10 V, DC offset 2: 6 V, ion energy: 10 V, aperture: 20 V, TOF tube: 4600 V, reflectron: 1000 V, microchannel plate gain: 1600 V, Timepix: −2200 V, and TOF cycles: 5000–10,000

**FIGURE 4:**
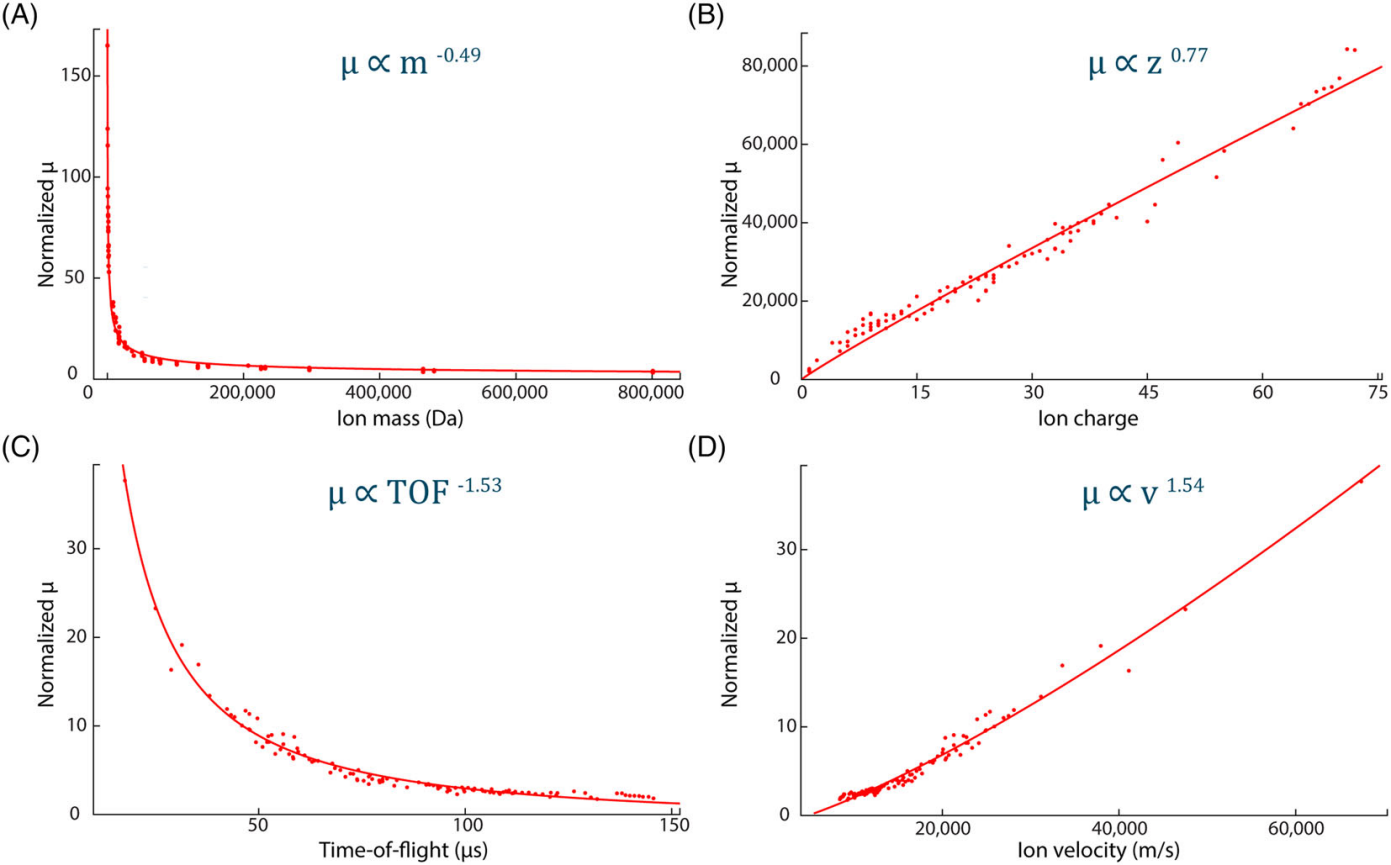
Influence of different ion properties ((A) ion mass, (B) ion charge, (C) TOF, and (D) ion velocity) on the average pixel cluster area (in pixels, *μ*). *μ* has been normalized by a factor of *z* ^0.77^ for (A), *m* ^−0.49^ for (B), and *m* ^0.28^ for (C) and (D)

Figure 3A,B are the 3D graphs, in which *μ* is plotted between *m* and *z*, and *m* and *TOF*, respectively. 3D curve fitting yields the values of 0.28, 1.54, −0.49, and 0.77 for the power constants *a*, *b*, *c*, and *d*, respectively. Figures 4 and S3 demonstrate the dependency of normalized *μ* on *m* (*R* ^2^ = 0.987), *z* (*R* ^2^ = 0.982), *TOF* (*R* ^2^ = 0.976), *v* (*R* ^2^ = 0.978), *m/z* (*R* ^2^ = 0.979), and ion impact energy (*E* = *zeV*; *R* ^2^ = 0.982). The detector performance has shown to improve with an increase in *q*, *v*, and *E*, as well as with a decrease in *m*, *m/z*, and *TOF* of the ion beam, which is in good agreement with the previous studies. However, the values of the exponents of mass and velocity appear to be different from the earlier studies, which can be attributed to the difference in the curve fitting approach. In previous works, in order to separate out the dependence of *γ* on *m* and *v*, *γ* has been plotted as *γ*_red_ = *γ*/*m*, called as reduced secondary electron yield, and fitted to a simple power law *γ*_red_ = *k*_5_ *v* ^*f*^ ^24^, ^25^, ^26^ or exponential function *γ*_red_ = *k*_6_ *e* ^*gv*^,^22^ where *k*_5_ and *k*_6_ are constants of proportionality, and *f* and *g* are fit parameters. Here, instead of assuming a linear dependence of *m* on *γ*, 3D curve fitting method was employed, which yields *γ* or *μ* ∝ *m* ^0.28^ *v* ^1.54^ or *m* ^−0.49^ *q* ^0.77^. For validating the derived function, the deviation of the experimentally determined *γ* value from Smith's data^25^ from their (2.6 × 10^−18^ *mv* ^3.1^) and our (1.04 × 10^−8^ *m* ^0.28^ *v* ^1.54^) fit functions were compared (Figure S4). Interestingly, our function fits better to the Smith data with an RMS error of 0.203 than their fit (RMS error = 0.247). Note that the *μ* to *γ* conversion was performed by calculating the ratio of *k*_0_ to *k*_4_, fitting *m* ^0.28^ *v* ^1.54^ to the *μ* data from this study and the *γ* values from Smith's group.

### 3.3 Influence of ion optics on MCP performance

**FIGURE 5:**
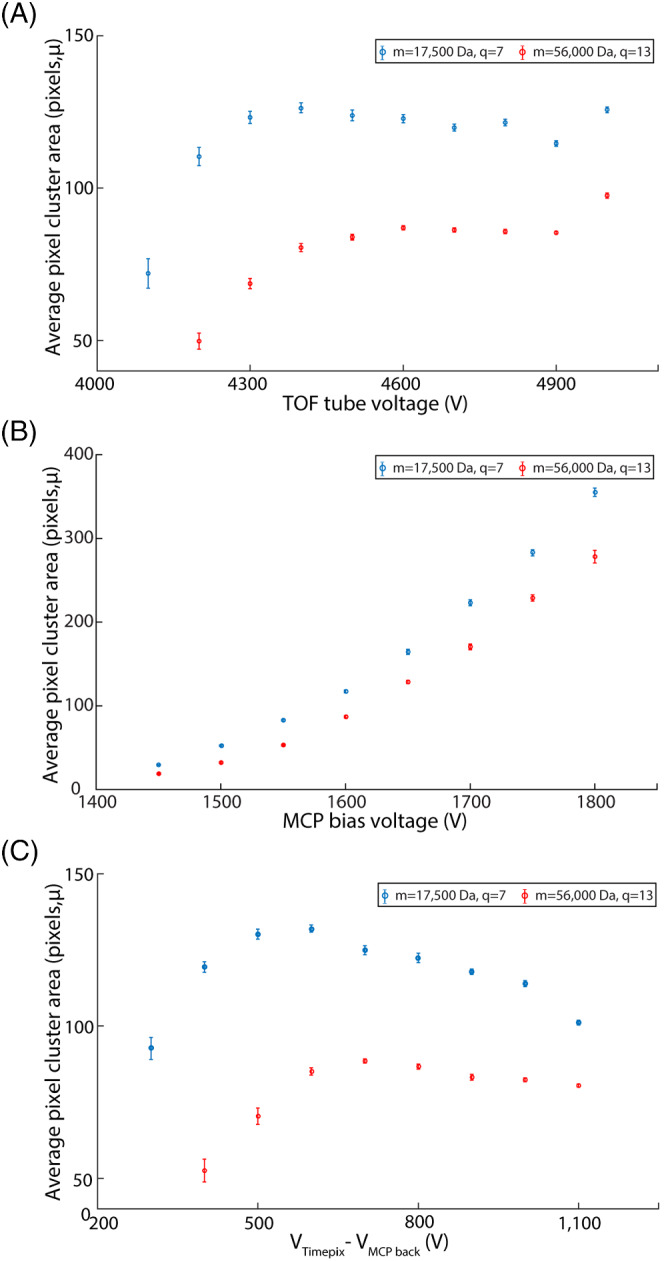
Effect of different ion optics parameters ((A) TOF tube voltage, (B) MCP bias voltage, and (C) voltage difference between MCP back plate and TPX detector [*V*_TPX–MCP back_]) on the average pixel cluster area (in pixels, *μ*). Red and blue traces correspond to concanavalin A dimer [M + 13H]^13+^ and holo‐myoglobin [M + 7H]^7+^ ions, respectively

We next investigated the influence of ion optics on the MCP detection efficiency. In our previous work, we have shown that the voltage settings “ion energy” and “RF DC offset voltage of first hexapole” have an influence on the axial ion energy of the ion beam, while “TOF tube voltage” affects the orthogonal ion energy.^32^ An increase in ion to electron conversion efficiency has been expected with a raise in orthogonal voltage as per Equation (5). Figure 5A shows the increasing trend of *μ* with the orthogonal TOF tube voltage. As per Equation (5), a power function of 0.77 was expected to fit with the TOF tube voltage–*μ* curve. However, the limited control over the TOF tube voltage (4000 to 5000 V) allows a change in velocity of the myoglobin [M + 7H]^7+^ ions from 17.6 to 19.6 km/s and concanavalin A dimer [M + 13H]^13+^ ions from 13.4 to 14.9 km/s, which covers only a very small portion of the velocity range shown in Figure 4D. Therefore, it is difficult to fit a power function to TOF tube voltage–*μ* curve with the limited number of data points. As expected, *μ* value remains unaffected by voltage settings “RF DC offset of first hexapole” and “ion energy,” because both these voltages contribute to the total energy of the ion beam only through the axial velocity component, which is parallel to the detection plane.

As discussed in the previous section, a higher MCP bias voltage increases the number of secondary electrons generated in each amplification step within the MCP and thus raises *μ* (Figure 5B). Figure 5B fits to an exponential function as expected. An increase in MCP bias voltage enhances the MCP output signal quality to some extent for high mass species. However, the independency of the ion to electron conversion factor on the MCP bias voltage still limits the detection of large slow moving ions.

*V*_TPX–MCP back_, the potential gradient between MCP back plate and TPX detector, is one of the parameters that has an influence on the overall detector performance. Figure 5C shows the dependence of *μ* on the *V*_TPX–MCP back_. At a lower *V*_TPX–MCP back_, the potential gradient is not strong enough to accelerate and focus the electron clouds from the MCP to the TPX at the low voltage. Most importantly, the charge deposited into a single pixel by the electron clouds is not adequate for the activation of TPX pixels. A higher amount of charge is deposited with an increase in TPX voltage, which leads to an increase in *μ*. However, after attaining a maximum value, *μ* tends to decrease due to the space charge effect. At a higher *V*_TPX–MCP back_, electrons are accelerated much faster towards the TPX detector, providing less time for the electron cloud to expand, which leads to the activation of less number of TPX pixels. The space‐charge‐driven expanded shape of the electron cloud was expected to fit a cosine distribution.^50^, ^51^

Understanding the influence of ion optics on MCP detector efficiency or electron cloud footprint size is crucial in the context of using an MCP as an image intensifier when combining with position readout systems such as charge coupled device (CCD), resistive anode encoder, delay line detector, discrete anode array detector, wedge and stripe anodes, hybrid active pixel detector (Medipix/Timepix, Pixel Imaging Mass Spectrometry [PImMS] camera, etc.) for the imaging of low energy photons (soft X‐ray, UV and visible), alpha particles, ions, and neutrons in various applications.^21^, ^52^, ^53^, ^54^, ^55^, ^56^ Optimization of the event charge footprint is required to achieve high spatial resolution and appropriate image linearity. A narrow charge cloud can cause image distortion, whereas excessive spreading of the charge footprint leads to distortion at the image edges. It was observed that the TOF tube voltage, MCP bias voltage, and potential gradient between back plate of the MCP and TPX can be used to fine tune the electron cloud footprint to achieve optimum image quality. These results are in good agreement with the previous studies conducted on the MCP electron charge cloud analysis using MCP in combination with split‐strip anode, cross‐strip anode, phosphor screen‐CCD assembly, and discrete anode array, where they have shown that the charge cloud can be described as a function of MCP bias voltage, acceleration bias voltage, and distance between the back plate of the MCP and position readout system.^57^, ^58^, ^59^, ^60^

## 4 CONCLUSION

This study comprises the comprehensive analysis of MCP detector performance in terms of ion properties and ion optics parameters for high mass multiply charged non‐covalent protein/protein complex ions that encompasses a wide mass range from 195 to 802,000 Da. The utilization of a TPX detector combined with the dual MCP stack allowed the characterization of MCP performance by analyzing the footprints of secondary electron clouds generated by the MCP on TPX corresponding to each individual ion events. Oversampling each individual ion events by a number of pixels for a number of TOF cycles makes this as the best method to examine the MCP response. The MCP detector performance is shown to improve with an increase in ion charge, velocity, and energy and deteriorates with an increase in ion mass, *m/z*, and TOF. The dependence of ion optical parameters such as TOF tube voltage, MCP bias voltage, and potential gradient between back plate of the MCP and TPX on the MCP charge cloud footprint can be utilized to achieve optimum image quality when MCP is used in imaging systems, as well as to improve native macromolecular ion detection efficiency in the high mass low velocity regime.

## Supporting information


**Figure S1.** Schematic of the ion detection mechanism in MCP‐TPX detection assembly. A number of electrons are ejected from the front MCP plate upon the impact of the ions. These electrons generate more electrons that are accelerated to the back MCP plate based on the MCP bias voltage. The TPX detector positioned behind the back MCP measures the arrival time, position and size of the emitted electron pulses that span multiple pixels.
**Figure S2**. TOF to m/z conversion curve, plotted by comparing LCT measured TOF data with the Orbitrap m/z spectrum of each sample. Calibration was performed by spraying 16 samples that encompasses the following mass range; m = 195 to 802,000 Da. All LCT TOF data were collected using the following voltage settings; TOF tube: 4,600 V, reflectron: 1,000 V, MCP gain: 1,600 V and TPX: −2,200 V.
**Figure S3**. Influence of different ion properties ((a) ion energy (b) m/z) on the average pixel cluster area (in pixels, μ). μ is normalized by a factor of m^−49^ for (a) and m^0.28^ for (b).
**Figure S4**. Secondary electron yield plotted as a function of ion velocity. The data shown in blue corresponds to the experimentally measured γ values from Smith's group.^1^ Orange and grey markers are the γ values calculated using Smith's (2.6 × 10^−18^ mv^3.1^) and our (1.04 × 10^−8^ m^0.28^v^1.54^) fit functions, respectively.

## Data Availability

Data are available on request from the authors.

## References

[1] Fenn JB , Mann M , Meng CK , Wong SF , Whitehouse CM . Electrospray ionization for mass spectrometry of large biomolecules. Science. 1989;246(4926):64‐71. doi:10.1126/science.2675315 2675315

[2] Karas M , Bachmann D , Bahr U , Hillenkamp F . Matrix‐assisted ultraviolet laser desorption of non‐volatile compounds. Int J Mass Spectrom. 1987;78:53‐68. doi:10.1016/0168-1176(87)87041-6

[3] Mirza UA , Cohen SL , Chait BT . Heat‐induced conformational changes in proteins studied by electrospray ionization mass spectrometry. Anal Chem. 1993;65(1):1‐6. doi:10.1021/ac00049a003 8380538

[4] Wenzel RJ , Matter U , Schultheis L , Zenobi R . Analysis of megadalton ions using cryodetection MALDI time‐of‐flight mass spectrometry. Anal Chem. 2005;77(14):4329‐4337. doi:10.1021/ac0482054 16013843

[5] Weidmann S , Barylyuk K , Nespovitaya N , Mädler S , Zenobi R . A new, modular mass calibrant for high‐mass MALDI‐MS. Anal Chem. 2013;85(6):3425‐3432. doi:10.1021/ac400129h 23394312

[6] Wilm M , Mann M . Analytical properties of the nanoelectrospray ion source. Anal Chem. 1996;68(1):1‐8. doi:10.1021/ac9509519 8779426

[7] Nesatyy VJ . Mass spectrometry evaluation of the solution and gas‐phase binding properties of noncovalent protein complexes. Int J Mass Spectrom. 2002;221(2):147‐161. doi:10.1016/S1387-3806(02)00956-9

[8] Iavarone AT , Udekwu OA , Williams ER . Buffer loading for counteracting metal salt‐induced signal suppression in electrospray ionization. Anal Chem. 2004;76(14):3944‐3950. doi:10.1021/ac049724+ 15253628 PMC1343452

[9] Verkerk UH , Kebarle P . Ion‐ion and ion‐molecule reactions at the surface of proteins produced by nanospray. Information on the number of acidic residues and control of the number of ionized acidic and basic residues. J Am Soc Mass Spectrom. 2005;16(8):1325‐1341. doi:10.1016/j.jasms.2005.03.018 15979326

[10] Sobott F , Hernandez H , McCammon MG , Tito MA , Robinson CV . A tandem mass spectrometer for improved transmission and analysis of large macromolecular assemblies. Anal Chem. 2002;74(6):1402‐1407. doi:10.1021/ac0110552 11922310

[11] van den Heuvel RH , van Duijn E , Mazon H , et al. Improving the performance of a quadrupole time‐of‐flight instrument for macromolecular mass spectrometry. Anal Chem. 2006;78(21):7473‐7483. doi:10.1021/ac061039a 17073415

[12] Rose RJ , Damoc E , Denisov E , Makarov A , Heck AJ . High‐sensitivity Orbitrap mass analysis of intact macromolecular assemblies. Nat Methods. 2012;9(11):1084‐1086. doi:10.1038/nmeth.2208 23064518

[13] Sanglier S , Leize E , Dorsselaer A , Zal F . Comparative ESI‐MS study of ∼2.2 MDa native hemocyanins from deep‐sea and shore crabs: from protein oligomeric state to biotope. J Am Soc Mass Spectrom. 2003;14(5):419‐429. doi:10.1016/S1044-0305(03)00131-4 12745211

[14] Ilag LL , Videler H , McKay AR , et al. Heptameric (L12)_6_/L10 rather than canonical pentameric complexes are found by tandem MS of intact ribosomes from thermophilic bacteria. Proc Natl Acad Sci. 2005;102(23):8192‐8197. doi:10.1073/pnas.0502193102 15923259 PMC1149426

[15] Snijder J , Rose RJ , Veesler D , Johnson JE , Heck AJ . Studying 18 MDa virus assemblies with native mass spectrometry. Angew Chem Int Ed Engl. 2013;52(14):4020‐4023. doi:10.1002/anie.201210197 23450509 PMC3949431

[16] Leney AC , Heck AJ . Native mass spectrometry: what is in the name? J Am Soc Mass Spectrom. 2017;28(1):5‐13. doi:10.1007/s13361-016-1545-3 PMC5174146 27909974

[17] Guilhaus M . Special feature: tutorial. Principles and instrumentation in time‐of‐flight mass spectrometry. Physical and instrumental concepts. J Mass Spectrom. 1995;30(11):1519‐1532. doi:10.1002/jms.1190301102

[18] Standing K . Timing the flight of biomolecules: a personal perspective. Int J Mass Spectrom. 2000;200(1‐3):597‐610. doi:10.1016/S1387-3806(00)00355-9

[19] Wiza JL . Microchannel plate detectors. Nucl Instrum Methods. 1979;162(1‐3):587‐601. doi:10.1016/0029-554X(79)90734-1

[20] Matsuura S , Umebayashi S , Kusuyama Y , Natsume Y , Oba K . Compact MCP assemblies for mass spectrometers. Nucl Instrum Methods Phys Res, Sect A. 1995;363(1‐2):481‐484. doi:10.1016/0168-9002(95)00172-7

[21] Jungmann JH , Heeren RM . Detection systems for mass spectrometry imaging: a perspective on novel developments with a focus on active pixel detectors. Rapid Commun Mass Spectrom. 2013;27(1):1‐23. doi:10.1002/rcm.6418 23239313

[22] Geno P , Macfarlane R . Secondary electron emission induced by impact of low‐velocity molecular ions on a microchannel plate. Int J Mass Spectrom. 1989;92:195‐210. doi:10.1016/0168-1176(89)83028-9

[23] Meier R , Eberhardt P . Velocity and ion species dependence of the gain of microchannel plates. Int J Mass Spectrom. 1993;123(1):19‐27. doi:10.1016/0168-1176(93)87050-3

[24] Westmacott G , Frank M , Labov S , Benner W . Using a superconducting tunnel junction detector to measure the secondary electron emission efficiency for a microchannel plate detector bombarded by large molecular ions. Rapid Commun Mass Spectrom. 2000;14(19):1854‐1861. doi:10.1002/1097‐0231(20001015)14:19<1854::AID‐RCM102>3.0.CO;2‐M 11006596 10.1002/1097-0231(20001015)14:19<1854::AID-RCM102>3.0.CO;2-M

[25] Liu R , Li Q , Smith LM . Detection of large ions in time‐of‐flight mass spectrometry: effects of ion mass and acceleration voltage on microchannel plate detector response. J Am Soc Mass Spectrom. 2014;25(8):1374‐1383. doi:10.1007/s13361-014-0903-2 24789774 PMC4108536

[26] Chen X , Westphall MS , Smith LM . Mass spectrometric analysis of DNA mixtures: instrumental effects responsible for decreased sensitivity with increasing mass. Anal Chem. 2003;75(21):5944‐5952. doi:10.1021/ac030127h 14588036

[27] Beuhler R , Friedman L . Threshold studies of secondary electron emission induced by macro‐ion impact on solid surfaces. Nucl Instrum Methods. 1980;170(1‐3):309‐315. doi:10.1016/0029-554X(80)91031-9

[28] Beuhler RJ . A comparison of secondary electron yields from accelerated water cluster ions (*M/z* < 50 000) striking Al_2_O_3_ and copper surfaces. J Appl Phys. 1983;54(7):4118‐4126. doi:10.1063/1.332545

[29] Axelsson J , Parilis E , Reimann C , Sullivan P , Sundqvist B . Electron emission from conducting surfaces impacted by multiply‐charged polyatomic ions. Nucl Instrum Methods Phys Res, Sect B. 1995;101(4):343‐356.

[30] Westmacott G , Ens W , Standing K . Secondary ion and electron yield measurements for surfaces bombarded with large molecular ions. Nucl Instrum Methods Phys Res, Sect B. 1996;108(3):282‐289. doi:10.1016/0168-583X(95)01060-2

[31] Brunelle A , Chaurand P , Della‐Negra S , Le Beyec Y , Parilis E . Secondary electron emission yields from a CsI surface under impacts of large molecules at low velocities (5 × 10^3^–7 × 10^4^ ms^−1^). Rapid Commun Mass Spectrom. 1997;11(4):353‐362. doi:10.1002/(SICI)1097‐0231(19970228)11:4<353::AID‐RCM865>3.0.CO;2‐4

[32] Mathew A , Buijs R , Eijkel GB , et al. Ion imaging of native protein complexes using orthogonal time‐of‐flight mass spectrometry and a Timepix detector. J Am Soc Mass Spectrom. 2021;32(2):569‐580. doi:10.1021/jasms.0c00412 33439014 PMC7863068

[33] Llopart X , Ballabriga R , Campbell M , Tlustos L , Wong W . Timepix, a 65k programmable pixel readout chip for arrival time, energy and/or photon counting measurements. Nucl Instrum Meth A. 2007;581(1‐2):485‐494. doi:10.1016/j.nima.2007.08.079

[34] Ponchut C , Clément J , Rigal J‐M , et al. Photon‐counting X‐ray imaging at kilohertz frame rates. Nucl Instrum Methods Phys Res, Sect A. 2007;576(1):109‐112. doi:10.1016/j.nima.2007.01.131

[35] Platkevic M , Cermak P , Jakubek J , et al. Characterization of charge collection in various semiconductor sensors with energetic protons and Timepix device. In: 2011 IEEE Nuclear Science Symposium Conference Record. IEEE; 2011:4715‐4719.

[36] Whyntie T , Harrison M . Simulation and analysis of the LUCID experiment in the Low Earth Orbit radiation environment. In: Journal of Physics: Conference Series. IOP Publishing; 2014 p. 022038.

[37] Stoffle N , Pinsky L , Kroupa M , et al. Timepix‐based radiation environment monitor measurements aboard the International Space Station. Nucl Instrum Methods Phys Res, Sect A. 2015;782:143‐148. doi:10.1016/j.nima.2015.02.016

[38] Vallerga J , McPhate J , Tremsin A , Siegmund O . High‐resolution UV, alpha and neutron imaging with the Timepix CMOS readout. Nucl Instrum Meth A. 2008;591(1):151‐154. doi:10.1016/j.nima.2008.03.046

[39] Campbell M , Collaboration M . 10 years of the Medipix2 Collaboration. Nucl Instrum Meth A. 2011;633:S1‐S10. doi:10.1016/j.nima.2010.06.106

[40] Tremsin A , Vallerga J , McPhate J , Siegmund O . Optimization of high count rate event counting detector with Microchannel Plates and quad Timepix readout. Nucl Instrum Methods Phys Res, Sect A. 2015;787:20‐25. doi:10.1016/j.nima.2014.10.047

[41] Ballabriga R , Campbell M , Llopart X . Asic developments for radiation imaging applications: the Medipix and Timepix family. Nucl Instrum Methods Phys Res, Sect A. 2018;878:10‐23. doi:10.1016/j.nima.2017.07.029

[42] Bamberger C , Renz U , Bamberger A . Digital imaging mass spectrometry. J Am Soc Mass Spectrom. 2011;22(6):1079‐1087. doi:10.1007/s13361-011-0120-1 21953049

[43] Jungmann JH , MacAleese L , Visser J , Vrakking MJ , Heeren RM . High dynamic range bio‐molecular ion microscopy with the Timepix detector. Anal Chem. 2011;83(20):7888‐7894. doi:10.1021/ac2017629 21882854

[44] Kiss A , Smith DF , Jungmann JH , Heeren RM . Cluster secondary ion mass spectrometry microscope mode mass spectrometry imaging. Rapid Commun Mass Spectrom. 2013;27(24):2745‐2750. doi:10.1002/rcm.6719 24214859

[45] Soltwisch J , Goritz G , Jungmann JH , et al. MALDI mass spectrometry imaging in microscope mode with infrared lasers: bypassing the diffraction limits. Anal Chem. 2014;86(1):321‐325. doi:10.1021/ac403421v 24308447

[46] Ellis SR , Soltwisch J , Heeren RM . Time‐resolved imaging of the MALDI linear‐TOF ion cloud: direct visualization and exploitation of ion optical phenomena using a position‐ and time‐sensitive detector. J Am Soc Mass Spectrom. 2014;25(5):809‐819. doi:10.1007/s13361-014-0839-6 24658803

[47] Syed SU , Eijkel GB , Kistemaker P , et al. Experimental investigation of the 2D ion beam profile generated by an ESI octopole‐QMS system. J Am Soc Mass Spectrom. 2014;25(10):1780‐1787. doi:10.1007/s13361-014-0958-0 25113629

[48] Jungmann JH , Smith DF , Kiss A , MacAleese L , Buijs R , Heeren RMA . An in‐vacuum, pixelated detection system for mass spectrometric analysis and imaging of macromolecules. Int J Mass Spectrom. 2013;341:34‐44.

[49] Ellis SR , Jungmann JH , Smith DF , Soltwisch J , Heeren RM . Enhanced detection of high‐mass proteins by using an active pixel detector. Angew Chem Int Ed. 2013;52(43):11261‐11264. doi:10.1002/anie.201305501 24039122

[50] Yamamura Y , Muraoka K . Over‐cosine angular distributions of sputtered atoms at normal incidence. Nucl Instrum Methods Phys Res, Sect B. 1989;42(2):175‐181. doi:10.1016/0168-583X(89)90704-0

[51] Gudmundsson JT . Physics and technology of magnetron sputtering discharges. Plasma Sources Sci Technol. 2020;29(11):113001. doi:10.1088/1361-6595/abb7bd

[52] Mantus DS , Morrison GH . Ion image detection with a microchannel plate evaluated by using a charge coupled device camera. Anal Chem. 1990;62(11):1148‐1155. doi:10.1021/ac00210a011

[53] Michalet X , Siegmund O , Vallerga J , Jelinsky P , Millaud J , Weiss S . Detectors for single‐molecule fluorescence imaging and spectroscopy. J Mod Opt. 2007;54(2‐3):239‐281. doi:10.1080/09500340600769067 20157633 PMC2821066

[54] Jungmann JH , Heeren RM . Emerging technologies in mass spectrometry imaging. J Proteomics. 2012;75(16):5077‐5092. doi:10.1016/j.jprot.2012.03.022 22469858

[55] Tremsin A , Vallerga J . Unique capabilities and applications of Microchannel Plate (MCP) detectors with Medipix/Timepix readout. Radiat Meas. 2020;130:106228. doi:10.1016/j.radmeas.2019.106228

[56] Tremsin A , Vallerga J , Siegmund O . Overview of spatial and timing resolution of event counting detectors with microchannel plates. Nucl Instrum Methods Phys Res, Sect A. 2020;949:162768. doi:10.1016/j.nima.2019.162768

[57] Edgar ML , Kessel R , Lapington JS , Walton DM . Spatial charge cloud distribution of microchannel plates. Rev Sci Instrum. 1989;60(12):3673‐3680. doi:10.1063/1.1140473

[58] Tremsin A , Siegmund O . Spatial distribution of electron cloud footprints from microchannel plates: measurements and modeling. Rev Sci Instrum. 1999;70(8):3282‐3288. doi:10.1063/1.1149905

[59] Tremsin AS , Siegmund OH . Charge cloud asymmetry in detectors with biased MCPs. In: X‐Ray and Gamma‐Ray Instrumentation for Astronomy XII. Vol.4497. International Society for Optics and Photonics; 2002:127‐138.

[60] Saito M , Saito Y , Asamura K , Mukai T . Spatial charge cloud size of microchannel plates. Rev Sci Instrum. 2007;78(2):023302. doi:10.1063/1.2472595 17578104

