# Supplementary material for: Characterization of microchannel plate detector response for the detection of native multiply charged high mass single ions in orthogonal‐time‐of‐flight mass spectrometry using a Timepix detector
Source: J Mass Spectrom. 2022 Mar 28;57(4):e4820. doi: 10.1002/jms.4820 (PMC9287041; doi:10.1002/jms.4820)
Supplement: Supplementary file 1 — Figure S1. Schematic of the ion detection mechanism in MCP‐TPX detection assembly. A number of electrons are ejected from the front MCP plate upon the impact of the ions. These electrons generate more electrons that are accelerated to the back MCP plate based on the MCP bias voltage. The TPX detector positioned behind the back MCP measures the arrival time, position and size of the emitted electron pulses that span multiple pixels. Figure S2. TOF to m/z conversion curve, plotted by comparing LCT measured TOF data with the Orbitrap m/z spectrum of each sample. Calibration was performed by spraying 16 samples that encompasses the following mass range; m = 195 to 802,000 Da. All LCT TOF data were collected using the following voltage settings; TOF tube: 4,600 V, reflectron: 1,000 V, MCP gain: 1,600 V and TPX: −2,200 V. Figure S3. Influence of different ion properties ((a) ion energy (b) m/z) on the average pixel cluster area (in pixels, μ). μ is normalized by a factor of m−49 for (a) and m0.28 for (b). Figure S4. Secondary electron yield plotted as a function of ion velocity. The data shown in blue corresponds to the experimentally measured γ values from Smith's group.1 Orange and grey markers are the γ values calculated using Smith's (2.6 × 10^−18^ mv3.1) and our (1.04 × 10^−8^ m0.28v1.54) fit functions, respectively. [file JMS-57-0-s001.docx]

**Supplementary Material**

**Characterization of Microchannel Plate Detector Response for the Detection of Native Multiply Charged High Mass Single Ions in Orthogonal-Time-of-Flight Mass Spectrometry Using a Timepix Detector**

Anjusha Mathew^1^, Gert B. Eijkel^1^, Ian G. M. Anthony^1^, Shane R. Ellis^1,2 *^, and Ron M. A. Heeren^1*^

^1^Maastricht MultiModal Molecular Imaging (M4i) Institute, Division of Imaging Mass Spectrometry (IMS), Maastricht University, 6229 ER Maastricht, The Netherlands

^2^Molecular Horizons and School of Chemistry and Molecular Bioscience, University of Wollongong, NSW 2522, Australia

*To whom correspondence should be addressed:

r.heeren@maastrichtuniversity.nl

sellis@uow.edu.au


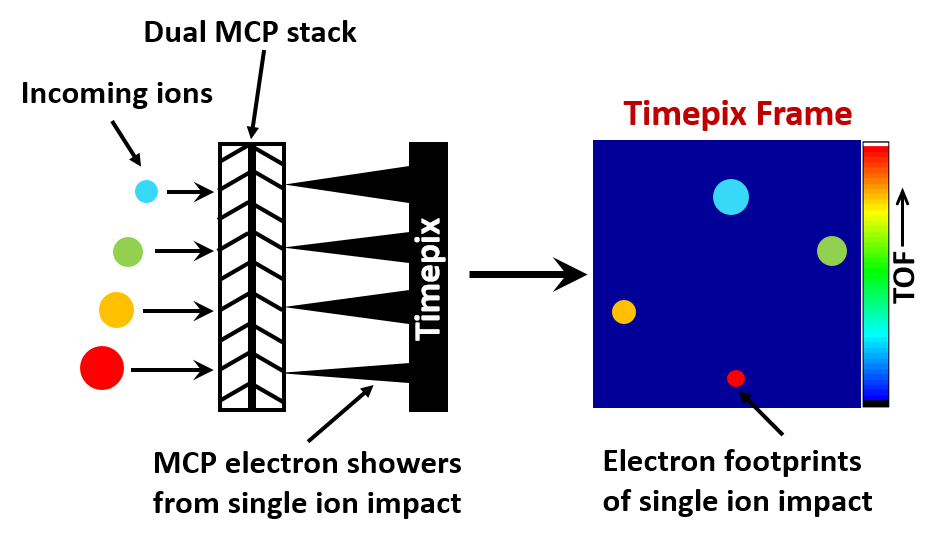


**Figure S1**. Schematic of the ion detection mechanism in MCP-TPX detection assembly. A number of electrons are ejected from the front MCP plate upon the impact of the ions. These electrons generate more electrons that are accelerated to the back MCP plate based on the MCP bias voltage. The TPX detector positioned behind the back MCP measures the arrival time, position and size of the emitted electron pulses that span multiple pixels.


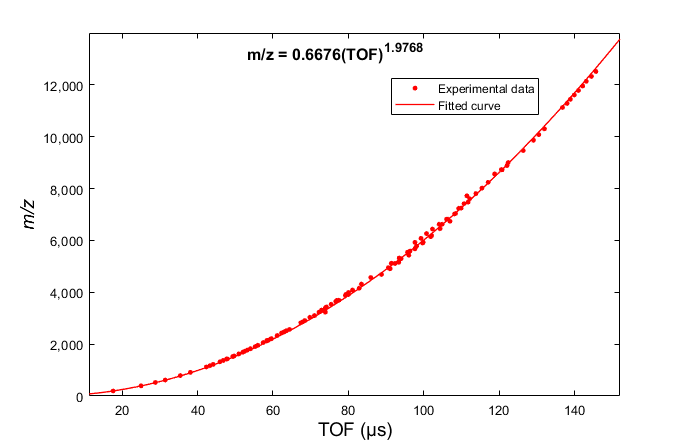


**Figure S2**. TOF to m/z conversion curve, plotted by comparing LCT measured TOF data with the Orbitrap m/z spectrum of each sample. Calibration was performed by spraying 16 samples that encompasses the following mass range; m = 195 to 802,000 Da. All LCT TOF data were collected using the following voltage settings; TOF tube: 4,600 V, reflectron: 1,000 V, MCP gain: 1,600 V and TPX: -2,200 V.


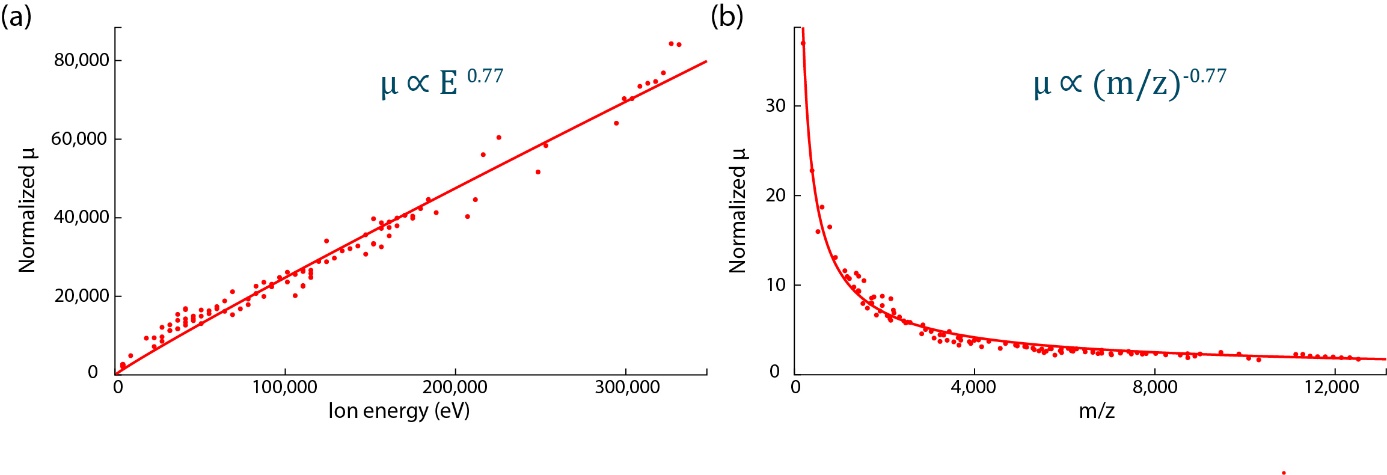


**Figure S3**. Influence of different ion properties ((a) ion energy (b) m/z) on the average pixel cluster area (in pixels, µ). µ is normalized by a factor of m^-49^ for (a) and m^0.28^ for (b).


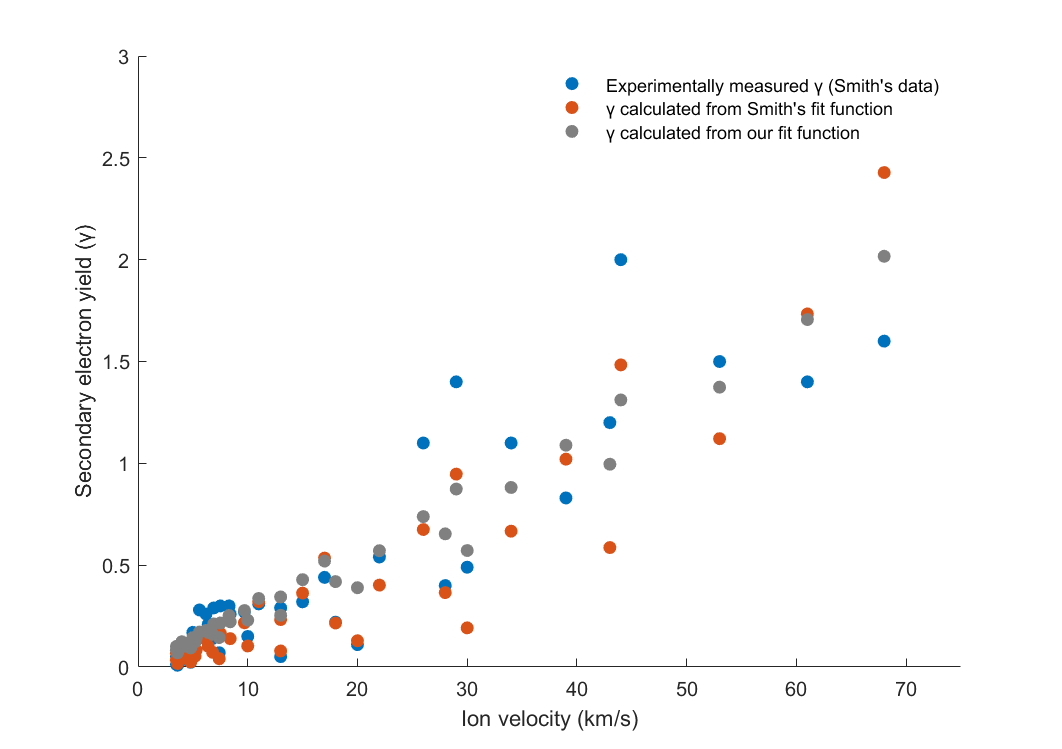


**Figure S4**. Secondary electron yield plotted as a function of ion velocity. The data shown in blue corresponds to the experimentally measured γ values from Smith’s group.^1^ Orange and grey markers are the γ values calculated using Smith’s (2.6 × 10^-18^ mv^3.1^) and our ( 1.04 × 10^-8^ m^0.28^v^1.54^) fit functions, respectively.

# **References**

1. Liu, R.; Li, Q.; Smith, L. M., Detection of large ions in time-of-flight mass spectrometry: effects of ion mass and acceleration voltage on microchannel plate detector response. *J Am Soc Mass Spectrom* **2014,** *25* (8), 1374-83.
